# The Prophet Not without Honor Save in Texas

**Published:** 1910-12

**Authors:** 


THE PROPHET NOT WITHOUT HONOR SAVE IN TEXAS.—The rational method of dealing with disease,—as with all evils,—is to prevent it.

No fact is better established than that alcohol causes insanity—and that alcoholic insanity is transmitted to "the third and fourth generation"—the family dies out in the fifth—which generation shows idiocy, imbecility, epilepsy and dementia. Meantime our asylums are overflowing with alcoholic—and other lunatics. Why not do something to prevent it, seeing that it is increasing out of all proportion to population; official statistics have been given time and again in the "Red Back," and I venture to mention again, here, now, that the cost to the taxpayer, for the care of the insane, keeps pace with this increase; for instance, the cost for the five years—1900 to 1905—was $3,555,000, or $707,000 (average) a year. Last year it was $938,358—just short of a million. The *San Antonio Express* says, editorially, that for the first time our insane are all cared for in the asylums of Texas, and that there are none in jail or poorhouse. The jails and poorhouses will fill up again, rapidly, as they always do, and about every four years additional buildings will have to be constructed. This takes place every few years. Governor Sayers during his last year told me that it was the proudest day of his life to say that not an insane person in Texas but what was then cared for. That was about six years ago. Since then the jails filled up, and last year an appropriation was made and expended for more room. Briefly. In 1860 the insane (50) cost $12,000. In 1900, $707,000. In 1904,

$784,000 (average, 1900-1905). In 1910—$938,358,—in round numbers;—an increase in five years of 35 per cent. At this rate, it will be but a short while before the insane will outnumber the sane, and the burden of their care become intolerable. Dr. Preston, in his report, says: "They will turn themselves out and lock us in."

Seeing that we have an anti-prohibition administration for the next four years, we can hardly hope for legislation to cut off,—even partly,—the source and cause of this dreadful increase, there remain but two courses to pursue: sterilize them—as advocated by this JOURNAL for years, and by Dr. Bogart, of Indiana, the author of the Indiana Sterilization Act, by Drs. Sharp, of Indiana, and Belfield, of Chicago; Carrington, of Virginia, and others (see Dr. Bogart's paper in this issue), or *operate on the stomach* of the *original* drunkard—"begin with the grandfather."

For the first mentioned measure, I intend to endeavor to secure the passage of an act like that of Indiana and Oregon (Dr. Bogart's bill). For the second, I recommend Dr. Kenney's gastro-enterostomy. Dr. Kenney will send you a reprint of his article, on request. It is the most important paper that has appeared on any subject; of equal importance with Ehrlich's. A discovery of far-reaching importance, not only to the drunkard operated on and cured, but as a prophylactic—that will *cut off the descent* of alcoholic insanity in the third, fourth and all succeeding generations! It was of this paper I intended to write when I wrote the caption of this article. The great metropolitan dailies of New York, Chicago and other Northern cities announced the discovery under scare headlines—commented on it editorially and reproduced it in full! They grasped at once the value and far-reaching importance of the measure,—*but not a paper in Texas* mentioned it. I have before me a copy of the *New York Times,* the foremost newspaper in the United States, of Sunday, October 30th, which contains the article in full. It has thus gone around the world, and will doubtless be appreciated—everywhere,—except in Texas.

